# The Choice before Us

**Published:** 1920-06-19

**Authors:** 


### The Hospital

The Workers' Newspaper of Administrative Medicine and Institutional Life, Administration, National Insurance and Health.

No. 1776, Vol. LXVIII. SATURDAY, JUNE 19, 1920. PRICE TWOPENCE

### THE CHOICE BEFORE US.

Suffering, as we all are, from the high cost of the necessaries of life, it is surprising that the difficulties of our hospitals (supported by voluntary contributions) have not been more fully realised by the general public. Not only are the maintenance charges tremendously increased, but the burdens thrown by the war on hospital supporters in the past have made it impossible for many of these to continue subscriptions, at any rate on the former generous scale. Any list of subscribers to a general hospital contains the names of many who have fixed incomes, and who for this reason have become the "new poor." It being manifestly impossible for these to live, and give, as in pre-war days, the result must be a falling off in subscriptions from old supporters. Those—and they are many—who are richer and better able to give than ever before, and those whose incomes rise with the cost of living, do not seem to have realised their responsibility to make up this deficit, and to increase the income of each hospital till it is sufficient adequately to maintain this splendid service to the nation. Yet in some way increased support must be forthcoming if hospitals are to continue to supply the needs of an ever-growing population for medical treatment and relief which cannot be given in humble homes. A moment's thought should be sufficient to bring home the urgency of the situation.

The hospitals of this country, built and maintained by voluntary aid, are a magnificent tribute to the sympathy and generosity of English men and women. The story—if it could be told—of what these institutions have achieved in the relief of suffering would be heart-stirring. All those who are brought into touch with the poor—especially in densely populated centres—speak, and cannot speak too highly, of the services rendered day and night to those who, but for our hospitals, would be compelled to suffer without relief or cure.

In some way then, greatly increased funds must be provided without delay. That the State should provide these funds, taking over the hospitals wholly or in part, is under consideration at the present time. Is this solution either desirable or necessary? Is it desirable, in view of the burden of taxation both Imperial and local, to make it still heavier by adding this costly service, hitherto rendered freely and without constraint? If all hospitals and institutions of like nature passed under Government control and were State supported, or even if they came under the county councils, and became chargeable to the rates, it is uncertain whether increased efficiency would result. There are many who dread the effect of destroying the voluntary basis lest this service and the spirit in which it is rendered be wholly or partially lost. There is a distinct danger that Government control would prove soulless and mechanical, in comparison with the present voluntary system.

Is the State hospital desirable from the point of view of economy? Experience of State Departments gives no hope of this, for the great incentive to economy is lost as soon as the pocket of the tax-payer or rate-payer can be tapped at will. Is it desirable when it would put an end to a system which calls forth so splendid a response and which makes so widespread an appeal to the better side of human nature? If the appeals now made to the general public on Hospital Sunday, and the collections made in the streets should cease, would this be no loss to our nation, and would it have no adverse effect on national character?

Let there be no mistake, once the State becomes wholly responsible, and the money required is raised by taxation—in any form—voluntary offerings will cease, for the spirit that prompts them now will be killed by compulsion. And if not desirable, is it necessary? Certainly not, if all will give according to their means. But if the present system is to be maintained it can only be by increased effort and by additional sacrifice.

Whether that effort and that sacrifice will be made, it is for the people of this country to decide, and to decide quickly. They must choose which it shall be, for there is no other alternative, and next week in its Hospital Sunday number it will be the wholehearted endeavour of this Journal clearly to outline a scheme whereby we believe that a first solution of the hospital crisis may yet be initiated by a co-ordination of the tried principles of the voluntary system.

